# Effects of Speech Clarity on Recognition Memory for Spoken Sentences

**DOI:** 10.1371/journal.pone.0043753

**Published:** 2012-09-07

**Authors:** Kristin J. Van Engen, Bharath Chandrasekaran, Rajka Smiljanic

**Affiliations:** 1 Department of Linguistics, University of Texas at Austin, Austin, Texas, United States of America; 2 Department of Communication Sciences and Disorders, University of Texas at Austin, Austin, Texas, United States of America; 3 Department of Psychology, Institute for Neuroscience, Center for Perceptual Systems, University of Texas at Austin, Austin, Texas, United States of America; University Of Cambridge, United Kingdom

## Abstract

Extensive research shows that inter-talker variability (i.e., changing the talker) affects recognition memory for speech signals. However, relatively little is known about the consequences of *intra-talker* variability (i.e. changes in speaking style within a talker) on the encoding of speech signals in memory. It is well established that speakers can modulate the characteristics of their own speech and produce a listener-oriented, intelligibility-enhancing speaking style in response to communication demands (e.g., when speaking to listeners with hearing impairment or non-native speakers of the language). Here we conducted two experiments to examine the role of speaking style variation in spoken language processing. First, we examined the extent to which clear speech provided benefits in challenging listening environments (i.e. speech-in-noise). Second, we compared recognition memory for sentences produced in conversational and clear speaking styles. In both experiments, semantically normal and anomalous sentences were included to investigate the role of higher-level linguistic information in the processing of speaking style variability. The results show that acoustic-phonetic modifications implemented in listener-oriented speech lead to improved speech recognition in challenging listening conditions and, crucially, to a substantial enhancement in recognition memory for sentences.

## Introduction

Spoken language contains information both about the content of a message and about the speaker of that message. Content is composed of several levels of linguistic information: sounds (phonological information), word-forming units (morphological information), combinations of words into sentences (syntactic information), and the meanings of words and word combinations (semantic information). The same auditory signal conveying all of this linguistic information also carries a wealth of information about the speaker: social (e.g., regional or social dialect features), affective (e.g., whether the person is happy, sad, excited, fatigued etc.), and personal (e.g., sex, age, as well as the size and shape of the vocal tract) [1], [2], [3], [4], [5], [6], [7], [8], [9].

Traditionally, the perception of linguistic content has been studied separately from the indexical properties of talkers. The emphasis in this line of work has been on how abstract linguistic units can be extracted from the immense variability in the speech signal. This abstractionist approach has been supported by a number of neuroscientific studies, which have shown that these two types of information are processed differently in the brain [10], [11], [12], [13], [14], [15]. For example, individuals with language deficits following a stroke do not show concomitant deficits in identifying speakers. Similarly, individuals with a neurological deficit that affects voice perception (phonoagnosia) show normal language comprehension skills. The finding that indexical and lexical information are dissociable is consistent with abstractionist accounts.

In contrast to abstractionist models, episodic approaches to speech processing contend that linguistic and indexical information are encoded and stored together in memory. These approaches have also been supported by a number of behavioral and neural studies showing that linguistic and indexical information are functionally integrated during speech processing [16], [17], [18], [19], [20], [21], [22], [23]. These studies show that properties of a talker's voice affect the processing of linguistic content in an utterance. For example, the recognition of words presented in noise is enhanced when listeners are familiar with the talker relative to words produced by an unfamiliar talker—an advantage that emerged for testing 5 minutes after exposure, but also up to a whole week after exposure [18]. Similarly, recognition memory in a continuous list of words has been shown to be more robust for words repeated in the same voice relative to a new voice [22].

By showing that talker variability affects recognition memory for words, these studies demonstrate the importance of indexical information in the processing of linguistic information. However, the focus of such studies has been on variability across talkers. In contrast, very little is known about the effects of speaking style changes by an individual speaker on the encoding of speech in memory. Extensive previous research has shown that speakers are able to enhance the intelligibility of their speech when asked to speak as if they are communicating with someone who is having difficulty accessing or understanding linguistic information. This intelligibility-enhancing speaking style (“clear speech” hereafter) is characterized by a number of acoustic/articulatory adjustments, including a decrease in speaking rate (both in terms of added pauses and in terms of increased duration of phonetic segments), increased dynamic pitch range, increased amplitude, more salient stop consonant releases, greater intensity of non-silent portions of consonants such as bursts and frication, and increased energy in the 1000–3000 Hz frequency range [24], [25], [26], [27], [28], [29], [30], [31], [32] (for a review, see [33]). In addition, it has been demonstrated that the distinctiveness of language-specific phonological vowel and consonant contrasts as well as of prosodic properties is enhanced in clear speech [25], [32], [34], [35], [36], [37], [38]. Together, these conversational-to-clear speech adjustments increase intelligibility, albeit to different degrees, for a wide range of listener populations, including normal hearing listeners [39], listeners with hearing impairment [40], [41], elderly listeners [42], non-native speakers of the target language [35], and children with and without learning disabilities [24]. As far as we know, however, no study has examined the effect of this type of intelligibility variability on recognition memory for linguistic content. Given that speakers constantly modify their speech during everyday communication in response to changing communication demands, it is of interest to examine the extent to which such changes impact memory for sentences.

This investigation of the effects of speech signal clarity on the robustness of memory representations also contributes to ongoing discussions in the literature on speech processing by aging and/or hearing-impaired adults. The “effortfulness hypothesis” [43], introduced by Rabbitt [44], [45], argues that perceptual processing in adverse listening situations may come at the cost of attentional resources that would otherwise be available for memory encoding [43], [46], [47], [48], [49], [50], [51], [52]. McCoy et al. (2005), for example, investigated recall of the final three words in a running word memory task by older adults with good hearing and poor hearing. All listeners were able to recall the final word with extremely high accuracy, indicating that they were all able to correctly perceive each word as it was presented. However, the adults with poor hearing recalled significantly fewer of the non-final words in word lists that lacked contextual constraint as opposed to word lists with high contextual constraint (i.e., where target words were predictable from the two prior words). It is argued that the higher orders of approximation may have facilitated target word recognition by increasing their likelihood, by decreasing the number of potential word candidates, and by aiding retrospective recognition of words that were unclear. Any of these mechanisms, they argue, might “reduce the perceptual burden on listeners' processing resources” and, therefore, aid recall.

In the present study, all listeners had normal hearing and the speech targets were not physically distorted or degraded, but their intelligibility was varied along the real-world dimension of within-talker speaking style changes. The effortfulness hypothesis leads to the prediction that greater attentional resources will be available for encoding the easier-to-perceive (i.e., clear) sentences in memory, leading to better recognition memory for clear speech versus conversational speech.

Specifically, this study investigated the extent to which changes in speaking style aimed at enhancing intelligibility affect memory for spoken language information. We tested such effects across two types of sentences: semantically anomalous and semantically normal (i.e., meaningful) sentences. Meaningful sentences presumably require less processing effort relative to anomalous sentences, and therefore were predicted to aid recognition memory and possibly modulate the effect of speaking style on recognition memory. Experiment 1 tested the intelligibility of all four sentence types as produced by a female native speaker of English. These sentences were presented to normal-hearing, young adult listeners in the presence of speech-shaped noise (i.e., white noise filtered so that its spectrum matches the long-term average spectrum of speech). The listening-in-noise paradigm was employed to avoid ceiling performance and to make the task difficult enough to reveal intelligibility differences between the two speaking styles. Listeners were asked to transcribe each sentence to the best of their ability. In Experiment 2, the sentences were presented in quiet to new listeners in a recognition memory experiment. For this task, listeners were exposed to a subset of conversational and clear sentences (40 total) and then tested on the full set (80 total), responding “old” (i.e., from the exposure set) or “new” to each item. We predicted that conditions in which perceptual effort is reduced, whether through acoustic-phonetic enhancements associated with clear speech or through the presence of semantic contextual information, would enhance recognition memory. Thus, the overall aim of these experiments was to investigate the extent to which within-talker variation in intelligibility affects the encoding of speech signals in memory. Our results indicate that indeed, such speaking style adjustments improve sentence intelligibility in noise (Experiment 1), and in turn, enhance their encoding in memory (Experiment 2). Thus, similar to the talker voice advantage, within-talker intelligibility modifications lead to better sentence recall.

## Methods and Results

### Ethics statement

All research protocols presented in this manuscript were approved by the Institutional Review Board at the University of Texas at Austin (approval #2010-11-0142).

### Experiment 1: Intelligibility of clear and conversational sentences

#### Participants

18 participants between the ages of 18 and 25 took part as listeners in Experiment 1. All participants were students at the University of Texas who were recruited via word of mouth or flyers posted on campus. All participants reported normal speech and hearing and were native, monolingual speakers of American English (i.e., they were born and raised in monolingual English households and local communities in which English is the primary language spoken, as reported in detailed background questionnaires). Potential participants who had significant exposure to another language before age 12 were not included. Participants provided written informed consent and were either paid or received course credit for their participation.

#### Stimuli

A 26-year-old female speaker of American English was recorded producing two sets of sentences: 1) the semantically anomalous sentences from the Syntactically Normal Sentence Test (SNST) [53] (e.g., *The wrong shot led the farm.*) and 2) semantically normal, i.e., meaningful, sentences generated by modifying sentences from the Basic English Lexicon (BEL) sentence materials [54] in order to closely match the SNST sentences in terms of syntax, length, and amount of keyword repetition within the set (e.g., *The grey mouse ate the cheese*). All sentences were produced in both clear and conversational speaking styles and contained four keywords each for intelligibility scoring. Recording took place in a sound-attenuated booth where sentences were presented to the speaker one at a time on a computer monitor. Following previous research [32], the two speaking styles were elicited with the following instructions: for conversational recordings, the speaker was asked to speak in a normal, conversational style, as if she was talking to someone familiar with her voice and speech patterns; for the clear speech recordings, the speaker was prompted to speak as though the listener was having a hard time understanding her, whether due to hearing difficulty or because the listener was a non-native speaker of English. Recordings were made using a Shure SM10A head-mounted microphone and a Marantz solid-state recorder (PMD670). Individual sentences were segmented from the long recording and equalized for RMS amplitude using Praat [55]. In order to verify that speaking style changes were implemented by the talker, the following acoustic measures were performed on all sentences that were used in the listening tests: duration, F0 range, mean F0, and average energy in the 1–3 kHz region.

40 sentences in each speaking style from each set were presented to listeners for assessment of intelligibility. Speech-shaped noise (SSN) was created for each sentence set (anomalous sentences in conversational speech; anomalous sentences in clear speech; meaningful sentences in conversational speech; meaningful sentences in clear speech) by filtering white noise to the long-term average spectrum of the full set of sentences. This approach was used to take into account any spectral differences across the sentence types and ensure that masking was consistent across the types.

#### Procedure

Participants first completed questionnaires about their language background. They were then seated in a sound-attenuated booth where they wore Sennheiser HD570 or Sony MDR-CD780 headphones. Instructions and stimuli were presented with EPrime [56]. In order to assess the relative intelligibility of clear and conversational speech produced by the speaker, each sentence was mixed with speech-shaped noise at a signal-to-noise ratio of 0 dB and then played to the participants, who were asked to transcribe as much of each sentence as they were able to understand. Each sentence was scored by the number of keywords correctly identified (4 per sentence) for a total of 160 keywords per sentence type. In order to be considered correct, no morphemes could be added to or deleted from the keywords, but homophones were accepted as a correct response. Listeners (nine per condition) heard a fully randomized set of either 80 semantically anomalous sentences (40 per speaking style) or 80 meaningful sentences (40 per speaking style). All stimuli were presented only once.

#### Results

**Figure 1:**
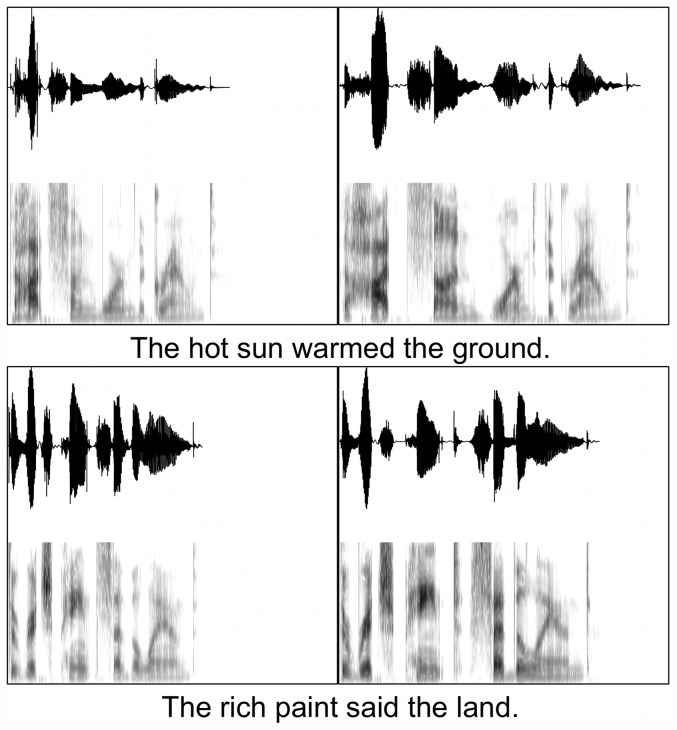
Waveforms and spectrograms of one meaningful sentence (top panels) and one anomalous sentences (bottom panels), each produced in both conversational (left panels) and clear (right panels) speaking styles. Each panel display represents 2.5 seconds.

Samples of both sentence types and speaking styles are shown in Figure 1, and average acoustic measures for each sentence set are given in Table 1. Paired t-tests confirmed that, for both sentence sets (anomalous and meaningful), clearly produced sentences had significantly longer durations than conversational speech. Clear sentences also had higher mean F0s (p<0.001 for both sentence sets) and larger F0 ranges (p<0.001 for both sentence sets). In the meaningful sentences, furthermore, clear speech was characterized by significantly greater energy in the 1–3 kHz range (p = .002). This trend was present but not significant for the anomalous sentences (p = .17). The analyses thus confirmed that the conversational and clear speech sentences differed in their acoustic-articulatory characteristics along the dimensions that are typically found in listener-oriented speaking style adaptations.

**Table 1:** Acoustic measures of sentence materials by speaking style and material type.

Mean (SD)	Clear speech: Anomalous	Conversational Speech: Anomalous	Clear speech: Meaningful	Conversational Speech: Meaningful
**Duration (s)**	2.87 (.44)	1.42 (.12)	3.21 (.35)	1.55 (.14)
**Average F0 (Hz)**	170.85 (7.91)	160.92 (8.68)	167.42 (7.90)	161.24 (8.85)
**F0 range (Hz)**	157.90 (91.02)	124.02 (100.97)	215.63 (122.69)	136.79 (108.25)
**Energy: 1–3 kHz**	23.22 (2.20)	23.10 (2.19)	22.17 (2.63)	22.61 (2.52)

**Figure 2:**
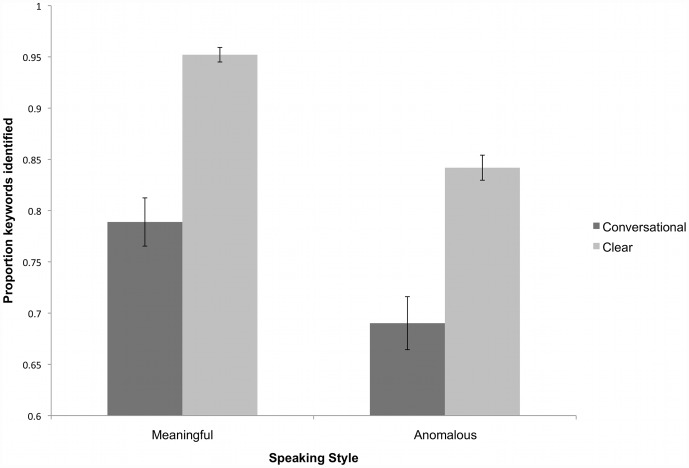
Average proportion of keywords identified from semantically anomalous and meaningful sentences produced in clear and conversational speaking styles. Error bars represent standard error.

The results of the intelligibility test are shown in Figure 2. For semantically anomalous sentences, listeners identified 69% of the keywords in conversational speech and 84% of the keywords in clear speech. For meaningful sentences, they identified 79% of the keywords in conversational speech and 95% of the keywords in clear speech. The intelligibility data were analyzed with a linear mixed effects logistic regression where keyword identification (i.e. correct or incorrect) was the dichotomous dependent variable. Subjects and Items were included in the model as random factors and Speaking Style, Semantic Content, and their interaction as fixed effects. Style was contrast coded (−.5, .5) such that negative beta values are associated with clear speech and positive beta values are associated with conversational speech. Similarly, Content was contrast coded (−.5, .5) such that negative beta values are associated with semantically anomalous sentences and positive values are associated with meaningful sentences. Analysis was performed using R [57]. The results of the regression are presented in Table 2.

**Table 2:** Results of the linear mixed effects logistic regression on intelligibility data for all sentences.

Fixed effects:	Estimate	Std. Error	z value	Pr(>|z|)
**(Intercept)**	3.3184	0.4619	7.184	6.75e-13 ***
**Semantics**	3.9740	0.9123	4.356	1.33e-05 ***
**Style**	−1.4941	0.1758	−8.496	<2e-16 ***
**Semantics:Style**	−1.0556	0.3284	−3.214	0.00131 **

The results show that the overall probability of correct keyword identification is significantly higher for meaningful versus anomalous sentences (p<0.001) and for clear versus conversational speech (p<0.001). Furthermore, there was a significant interaction between Speaking style and Semantic content (p = 0.001). The nature of this interaction was examined by performing mixed-effects logistic regressions on the Meaningful and Anomalous conditions separately. The results of these regressions are shown in Table 3 and Table 4. These regressions revealed that, while the effect of speaking style was a highly significant predictor of correct keyword identification for both types of sentences, the effect of style was greater (further from 0) for the meaningful sentences (β_anom_ = −.99; β_meaningful_ = −1.86).

**Table 3:** Results of the linear mixed effects logistic regression on intelligibility data for anomalous sentences.

Anomalous Sentences Fixed effects:	Estimate	Std. Error	z value	Pr(>|z|)
**(Intercept)**	1.3805	0.1364	10.122	<2e-16 ***
**Style**	−0.9903	0.2259	−4.383	1.17e-05 ***

**Table 4:** Results of the linear mixed effects logistic regression on intelligibility data for meaningful sentences.

Meaningful Sentences Fixed effects:	Estimate	Std. Error	z value	Pr(>|z|)
**(Intercept)**	8.0046	1.7574	4.555	5.24e-06 ***
**Style**	−1.8616	0.3759	−4.952	7.34e-07 ***

These results replicate previous studies that show that listener-oriented conversational-to-clear speech modifications enhance sentence intelligibility (see [33] for a review of the clear speech literature). Furthermore, the presence of semantic context significantly improved intelligibility overall, though listeners received a greater clear speech benefit for meaningful sentences than anomalous sentences. With these differences in intelligibility confirmed, Experiment 2 addresses the effects of such differences on sentence recognition memory.

### Experiment 2: Recognition memory for clear and conversational speech

#### Participants

33 young adults between the ages of 18 and 31 took part in Experiment 2: recognition memory for semantically anomalous sentences (n = 18, ages 18–31) or meaningful sentences (n = 15, ages 18–23). All participants were students at the University of Texas who were recruited via word of mouth or flyers posted on campus. No participant reported a history of speech, language, or hearing problems. All participants were native, monolingual speakers of American English (see criteria in Experiment 1) and none of them had participated in Experiment 1. All participants passed a hearing-screening test (1000, 2000, and 4000 Hz at 25 dB). They provided written informed consent and were either paid for their participation or received course credit.

#### Stimuli

The stimuli included a total of 160 semantically anomalous sentences or 160 meaningful sentences. The sentences were presented without noise. In order to confirm that the subsets of sentences used as old and new for recognition memory did not vary systematically in their intelligibility, the intelligibility data from Experiment 1 was further analyzed. Unpaired, 2-tailed t-tests were conducted to compare the intelligibility of the sentences that were to be used as new and old in the recognition memory experiments. These tests showed no significant difference between the intelligibility of old and new sentences.

#### Procedure

Participants first completed language background questionnaires. They were then seated in a sound-attenuated booth facing a computer monitor and wearing headphones. Instructions and stimuli were presented with EPrime [56], and listener responses were collected using a button box. During the exposure phase, listeners heard 40 unique sentences in random order and were instructed to try to commit them to memory. 20 of the sentences were presented in conversational speech, and 20 in clear speech. Listeners heard each sentence only one time, and sentences were separated by 500 ms of silence. At the end of the exposure phase, listeners were instructed that they would listen to another set of sentences. This time, they were instructed to indicate, using the button box, whether each sentence was new or old (from the exposure phase). All 40 of the exposure sentences were included, along with 40 new items (also half conversational and half clear). These 80 items were fully randomized for each participant, and they heard each one only once. At the end of the test phase, listeners were given the opportunity to take a break. They then completed the entire task a second time with 80 new sentences. This second block was included to ensure consistent performance across different sets of items.

#### Results

**Figure 3:**
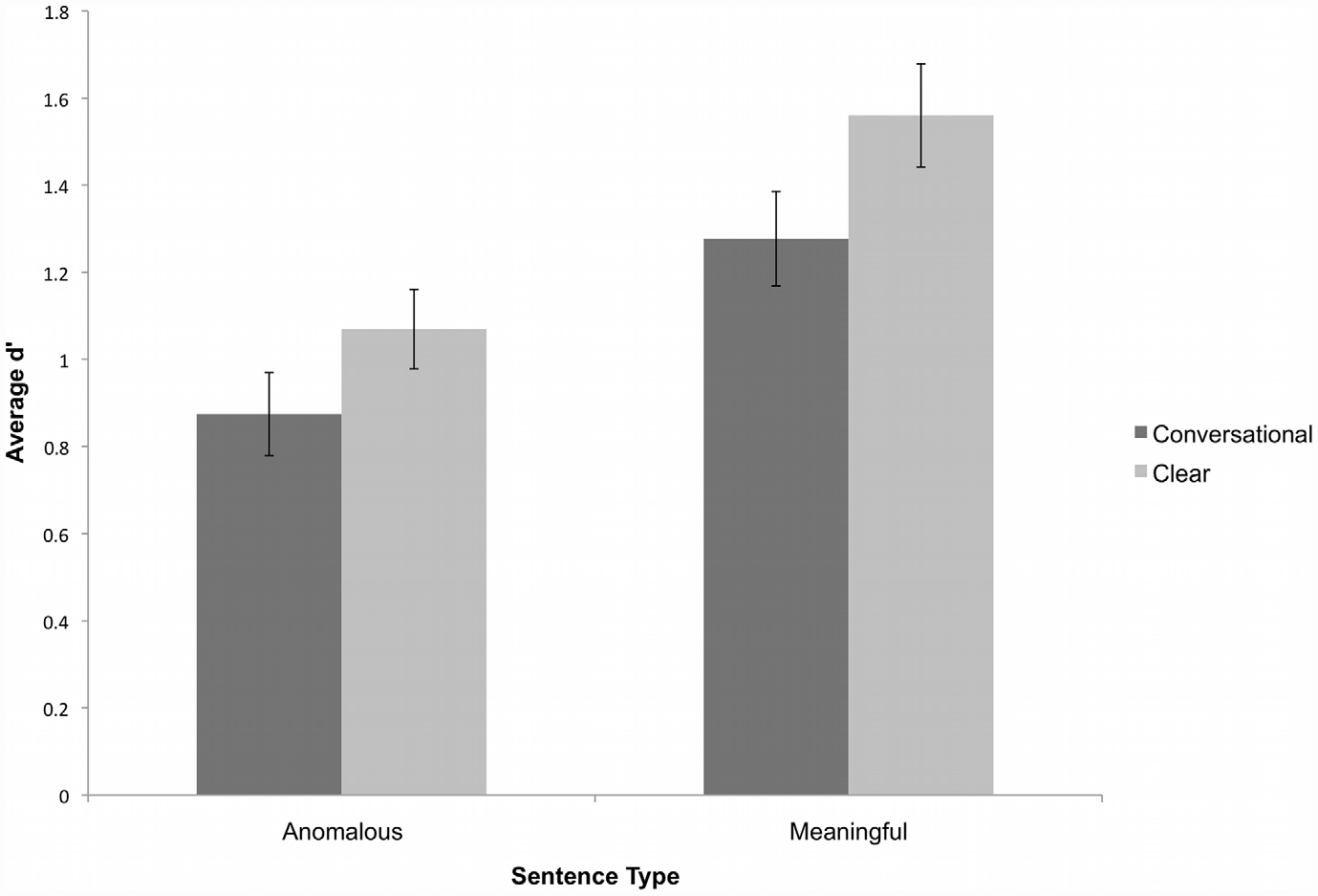
Average d′ scores in both testing blocks for semantically anomalous and meaningful sentences produced in clear and conversational speaking styles. Error bars represent standard error.

The recognition memory data was analyzed within a signal detection framework. To this end, d′ and C scores were computed for each participant to assess discrimination sensitivity and bias. d′ is calculated by subtracting the normalized probability of false alarms (identifying a new item as old) from the normalized probability of hits (identifying an old item as old). Those probabilities were then corrected to accommodate values of 0 and 1 in the d′ calculation [58]. Table 5 displays all uncorrected hit rates and false alarm rates as well as the calculated d′ and C scores. The average C scores across all conditions are positive, meaning participants were generally biased to respond “new” more often than “old.” This bias was stronger for speech produced in a clear style. The overall results of Experiment 2, presented as D′ scores, are shown in Figure 3.

**Table 5:** Calculated hit rates, false alarm rates, d′, and C values for the recognition memory test.

	Conversational Speech				Clear speech			
	Hit Rate	False Alarm Rate	d′	C	Hit Rate	False Alarm Rate	d′	C
**Anomalous Block 1**	0.67	0.31	0.96	0.02	0.64	0.21	1.19	0.23
**Anomalous Block 2**	0.63	0.34	0.95	0.04	0.56	0.22	1.24	0.27
**Meaningful Block 1**	0.69	0.25	1.16	0.08	0.70	0.15	1.56	0.26
**Meaningful Block 2**	0.73	0.25	1.39	0.04	0.64	0.13	1.56	0.39

D′ scores were submitted to a repeated measures ANOVA with Speaking Style (conversational or clear) and Block (1^st^ or 2^nd^) as within-subjects factors and Semantic Content (anomalous vs. meaningful) as a between-subjects factor. This analysis revealed main effects of Speaking style (F(1,31) = 8.975, p = .005) and Semantic content (F(1,31) = 13.489, p = .001), with better performance on semantically meaningful sentences and on sentences produced in a clear style. There was no significant effect of Block (first vs. second), and no significant interactions between Speaking style, Semantic content, and/or Block.

## Discussion

We examined the extent to which speaking style modifications facilitate recognition memory for spoken sentences. Experiment 1 evaluated the intelligibility of meaningful and semantically anomalous sentences spoken in clear and conversational styles. Experiment 2 examined listeners' recognition memory for these sentences. As predicted, acoustic-phonetic and semantic contextual enhancements resulted in better intelligibility, as evidenced by improved sentence recognition in noise (Experiment 1). Further, the intelligibility enhancement for clear speech was greater for meaningful sentences than for anomalous sentences. Importantly, the results demonstrated that clear speech sentences and meaningful sentences significantly improved recognition memory compared to conversational and semantically anomalous sentences (Experiment 2).

The results of Experiment 1 are consistent with previous studies showing that clear speech enhances intelligibility for listeners (see reviews in [33], [59]) and that semantic contextual information enhances the intelligibility of speech in noise [60], [61], [62], [63]. Furthermore, the enhancing effect of clear speech was significantly greater for meaningful sentences than for anomalous sentences, which indicates that these two factors independently improve intelligibility and mutually enhance the contributions of one another. Semantic contextual information and a clear speaking style thus benefit intelligibility in a cumulative manner through the speech processing system (cf. [60]).

Most importantly, this study showed that, in addition to being more intelligible than conversational speech, clear speech also led to better performance on a recognition memory task. The observed differences in recognition memory cannot be attributed to differences in whether the sentences were recognized correctly, because all sentences in the memory experiment were presented in quiet, rendering them intelligible to listeners. Rather, speaking style changes that enhanced intelligibility (as shown in Experiment 1) contributed to enhanced recognition memory (Experiment 2). It is worth noting that the enhanced recognition memory for clear speech was manifested largely in a lower rate of false alarm responses (see Table 4). This pattern of results has been shown in other studies of recognition memory (e.g., [64], [65], [66]) and has been interpreted as evidence for differences in the availability of distinctive features in memory for different types of stimuli [64]. In the present case, a greater number of distinctive features may be available to listeners in memory for clear speech versus conversational speech. In particular, we suggest that the exaggerated acoustic-phonetic cues in clear speech enhance memory traces for sentences produced in that style.

To understand how these enhanced memory traces might result in lowered false alarm rates, imagine (for simplicity's sake) that a participant has a single distinctive feature in memory for a given conversational sentence (CO1) and five distinctive features in memory for a given clear sentence (CL1). If either sentence is presented as a target (old) item during the recognition task, the person has a good chance of recognizing it as old, since people can identify items as old with very few distinctive features. If another conversational sentence (CO2) is presented as a distractor (new), however, and it happens to have a feature that is very similar to the feature in memory for CO1, then the person is likely to produce a false alarm since s/he has no other features in memory on which to base a rejection. In contrast, if another clear sentence (CL2) is presented as a distractor, it may have a feature very similar to one of the features in memory, but the person has four other features on which to base a correct rejection. (See Lamont et al. (2005) for a similar discussion.) In this way, the false alarm rate can be higher for conversational sentences while the hit rates are similar across sentence types. The present data do not allow us to speculate whether this memory enhancement occurs at the segmental, suprasegmental, lexical, or semantic level (or, most likely, through interactions at various levels).

The current results thus show that the beneficial effects of clear speech go beyond facilitating word identification and can also provide advantages in downstream processes such as encoding in memory. It remains to be determined what particular features of clear speech may underlie the observed improvements in recognition memory and whether these are the same features that contribute to enhancements in intelligibility. The acoustic analysis of the clear and conversational speech produced for this study showed several typical differences between conversational and clear speech: clear speech had longer duration, higher average F0 (corresponding to pitch), and greater energy in the 1–3 kHz range. It is important to note, however, that the exact articulatory-acoustic cues that contribute to the clear speech advantage remain rather elusive [67], [68], [69]. Research focus, thus, remains on finding the relevant acoustic-phonetic clear speech features and establishing their impact on intelligibility and recognition memory.

In addition to providing new evidence for the beneficial effects of clear speech on speech processing, this study extends previous work on the effects of speech signal variability on recognition memory. Specifically, where previous studies have shown that across-talker variability has significant effects on recognition memory for speech [18], [22], the present study shows that within-talker speaking style changes also significantly affect recognition memory. Since both the clear and conversationally produced sentences were fully intelligible to listeners in the memory experiment (no noise distortion), this result is generally compatible with accounts of speech processing that emphasize episodic encoding in memory.

The finding that clear speech led to better recognition memory than conversational speech is also in keeping with the effortfulness hypothesis [43], [70], which suggests that, by reducing the cognitive effort associated with perceptual speech processing, more processing resources will be available for encoding speech content in memory. Our results provide novel support for the hypothesis in that more easily recognized clear speech (as indicated by improved word recognition) was also encoded better in memory. The results suggest that, because clear speech requires less “effort” on the part of the listener, more processing resources could be recruited for retaining more information about the spoken sentences in memory.

The finding that the presence of semantic context significantly enhanced recognition memory is also in line with the effortfulness hypothesis. Previous studies have shown that processing meaningful stimuli leads to improvement in ‘chunking’ and recall [71], [72]. Presumably, semantically congruous sentences can be chunked into smaller memory units. This chunking reduces processing demands, leaving more resources available for memory encoding. In contrast, encoding semantically incongruous information as in the anomalous sentences likely requires more processing resources, which may lead to poorer memory encoding.

The current results additionally provide new evidence of the cumulative benefit of acoustic-phonetic and semantic contextual enhancements in naturally produced speech on memory encoding. That is, both sources of intelligibility variability significantly affect available processing resources and memory encoding. The results further suggest that both intelligibility and sentence recognition memory are shaped by the interplay of peripheral-auditory (clarity of the speech signal) and central-cognitive (semantic) factors. Future research needs to address the exact mechanism that underlies how processing resources are allocated in different tasks (e.g., word recognition vs. recognition memory) for speech of varying intelligibility.

There are several practical implications of these results. First, the results reported here suggest that the encoding of speech signals in memory may be affected by other common sources of variability in speech intelligibility, such as foreign accent, speech production impairment, and the presence of noise in the communicative environment – all cases where speech processing will require additional cognitive effort. Second, there are a number of listener populations for whom extra effort must regularly be expended in order to achieve perceptual success in the course of everyday speech communication. These groups include individuals with hearing impairment, auditory processing deficits, and cochlear implants, as well as older adults. Furthermore, noisy environments increase the level of perceptual effort required for individuals of all hearing abilities – a fact which may be particularly relevant for children learning in noisy classrooms. Our results suggest that perceptual success in these situations may come at the cost of processing resources that would otherwise be available for encoding the speech content in memory. It is important, therefore, that those who communicate regularly with these populations (e.g., hearing professionals, caretakers, teachers, etc.) be aware that apparent memory problems may, in fact, be rooted in perceptual difficulties, and further, that simply speaking clearly for such listeners can enhance not only the intelligibility of speech, but also a person's ability to encode it in memory.
